# A polymorphism of *HMGA1* protects against proliferative diabetic retinopathy by impairing HMGA1-induced VEGFA expression

**DOI:** 10.1038/srep39429

**Published:** 2016-12-19

**Authors:** Eusebio Chiefari, Valeria Ventura, Carmelo Capula, Giorgio Randazzo, Vincenzo Scorcia, Monica Fedele, Biagio Arcidiacono, Maria Teresa Nevolo, Francesco Luciano Bilotta, Michela Vitiello, Camillo Palmieri, Elio Gulletta, Alfredo Fusco, Daniela Foti, Raffaella Vero, Antonio Brunetti

**Affiliations:** 1 Department of Health Sciences, University “Magna Græcia” of Catanzaro, Catanzaro, Italy; 2 Operative Unit of Endocrinology and Diabetes, Hospital Pugliese-Ciaccio, Catanzaro, Italy; 3 Department of Clinical and Experimental Medicine, University “Magna Græcia” of Catanzaro, Catanzaro, Italy; 4 Institute of Experimental Endocrinology and Oncology, CNR, Napoli, Italy; 5 Department of Molecular Medicine and Medical Biotechnologies, University of Naples “Federico II”, Napoli, Italy

## Abstract

Diabetic retinopathy (DR) is a major complication of diabetes mellitus, and is the leading cause of blindness in working-age people. Usually, DR progresses from the asymptomatic non-proliferative DR that does not significantly alter vision, to proliferative DR (PDR), which can result in aberrant retinal neovessel formation and blindness. The High-Mobility-Group A1 (HMGA1) protein is a transcriptional master regulator of numerous genes, including metabolic and inflammatory genes, which, by modulating the expression of angiogenic factors, may induce retinal neovascularization, a hallmark of PDR. Herein, we examined the relationship between *HMGA1* rs139876191 variant and DR. Results revealed that patients with type 2 diabetes, who were carriers of the *HMGA1* rs139876191 variant had a significantly lower risk of developing PDR, compared to non-carrier diabetic patients. From a mechanistic point of view, our findings indicated that, by adversely affecting HMGA1 protein expression and function, the *HMGA1* rs139876191 variant played a key role in this protective mechanism by downregulating the expression of vascular endothelial growth factor A (VEGFA), a major activator of neovascularization in DR. These data provide new insights into the pathogenesis and progression of DR, and may offer opportunities for discovering novel biomarkers and therapeutic targets for diagnosis, prevention and treatment of PDR.

Diabetic retinopathy (DR) is the most common microangiopathic complication of diabetes mellitus, affecting over 30% of diabetic patients, and the leading cause of blindness among working-age adults in developed countries^1^,^2^. With the widespread diffusion of type 2 diabetes, the prevalence of DR is increasing worldwide along with rising health-care expenses and labor costs^2^. Thus, developing strategies to prevent and effectively treat DR is extremely important. Conceptually, DR consists of an early non-proliferative stage (NPDR) characterized by microaneurysms, dot and blot hemorrhages, retinal vascular leakage with exudate accumulation, and a more advanced, proliferative stage (PDR), in which visual loss can occur from either proliferation of new retinal vessels, or increased permeability of retinal blood vessels^3^. Several lines of evidence indicate that both increased vascular permeability and neovascularization in PDR may depend on the local production of angiogenic factors, inflammatory cytokines, chemokines and growth factors, in addition to components of the extracellular matrix, which will be substrates for endothelial migration^4^. In this context, the vascular endothelial growth factor-A (VEGFA), a major activator for angiogenesis^5^, is believed to play significant roles by inducing neovascularization and increasing permeability of retinal vessels^4^. In line with this, VEGFA expression is induced by hyperglycemia and hypoxia^1^, two hallmarks of diabetic complications, whereas levels of VEGFA are markedly increased in the vitreous of diabetic patients with active PDR^6^. However, despite many investigations, the underlying etiology of DR is largely unknown^7^, although genetic factors may contribute to both the occurrence and severity of this disease^8^. In this regard, the heritability estimates reported seem to be as high as 27% for NPDR and 50% for PDR^9^,^10^. Investigations using different approaches, including the more recent genome-wide association studies (GWAS), have been conducted, but success in identifying the genetic variants involved in DR has been limited^8^,^11^.

HMGA1 is an architectural transcription factor that acts as a dynamic regulator of chromatin structure and gene activation^12^. Defects in *HMGA1* gene and protein expression have been associated with insulin resistance and increased susceptibility to type 2 diabetes in humans and mice^13^,^14^,^15^, whereas protection against insulin resistance has been reported in transgenic mice overexpressing HMGA1^16^. Further evidence, implicating the *HMGA1* locus as one conferring high risk for the development of type 2 diabetes, has been provided recently by showing that a specific single-nucleotide insertion at position −13 of exon 6 of the *HMGA1* gene (IVS5–13insC; more precisely, c.136–14_136–13insC; rs139876191), significantly associates with type 2 diabetes in a transethnic meta-analysis^17^. Also, HMGA1 is a hypoxia-inducible factor that modulates the expression of several angiogenic proteins^18^,^19^,^20^, other than the production of cytokines, chemokines and adhesion molecules^21^ that, by triggering endothelial dysfunction via inflammation, may play a pathogenetic role in DR. Moreover, HMGA1 is highly expressed in murine retinas^22^, while cis-regulatory elements for HMGA1 are more abundant in promoters of genes preferentially expressed in retinal endothelial cells^23^.

Based on the above considerations, it was the aim of this study to evaluate the association of the *HMGA1* gene polymorphism rs139876191 with DR, and determine the role of this allele’s variant, if any, in the pathogenesis of this diabetic complication.

## Results

Demographic, anthropometric, clinical and biochemical features of enrolled patients are in Table 1. As assessed by non-parametric Mann-Whitney test, diabetic patients with PDR differed from diabetic control patients without DR in the following respects: higher HbA1c (*P* = 0.002), FPG (*P* = 0.004), systolic and diastolic BP (*P* < 0.001) and a higher prevalence of nephropathy (*P* = 0.015) (Table 1). In addition, patients in the PDR and NPDR groups had a significantly lower prevalence of hypoglycemic drug use (*P* < 0.001 and *P* = 0.006, respectively), a higher insulin use (*P* < 0.001 and *P* = 0.005, respectively) and a higher hypolipidemic drug treatment (*P* < 0.001 in both groups), as compared with diabetic control individuals without DR (Table 1).

### Case-control association of *HMGA1* rs139876191

The *HMGA1* rs139876191 was less common in diabetic patients with PDR than in those without PDR. Among the 436 patients with PDR, 20 (4.6%) were heterozygous and 1 (0.2%) was homozygous for the rs139876191 polymorphism (MAF 2.52%) (Table 2). In contrast, of the 587 diabetic patients with NPDR, 50 (8.5%) were heterozygous carriers (MAF 4.26%) and no subject was homozygous (Table 2). Similarly, among 936 diabetic control subjects without DR, 75 (8.0%) were heterozygous carriers (MAF 4.01%), and none was homozygous (Table 2). No significant deviation from Hardy-Weinberg equilibrium was detected (*P* = 0.16 for PDR cases; *P* = 0.28 for NPDR cases; *P* = 0.20 for controls). Logistic regression analysis with age and gender as covariates indicated that the presence of *HMGA1* rs139876191 was associated with over 40% [OR 0.573 (95% CI, 0.348–0.943), *P* = 0.029] lower risk of PDR (Table 2). The same relationship [OR 0.518 (95% CI, 0.309–0.868), *P* = 0.013] was observed when other covariates (i.e., duration of diabetes, hypertension, *HbA1c*, HDL-cholesterol, hypolipidemic and antihypertensive therapy) were included in the analysis (Table 2). In contrast, no association of the rs139876191 variant was observed with NPDR (Table 2), thus supporting the specific effect of this variant on PDR. It was interesting to note that during the study period, 38 diabetic patients initially classified as NPDR developed PDR, but only two of them were carriers of the rs139876191 variant.

### Association of the *HMGA1* rs139876191 with clinical and biochemical features

To determine whether the *HMGA1* rs139876191 was also associated with distinct clinical and biochemical characteristics, several quantitative and qualitative variables were evaluated in both carrier and non-carrier groups using multiple regression analysis (Table 3). Age, age at diagnosis of diabetes and BMI were similar in the two diabetic patient groups, and no significant differences were detectable among groups with regard to hypertension prevalence. No significant differences were also observed between these two groups concerning FPG levels, HbA1c, serum cholesterol (total, HDL-, LDL-cholesterol) and triglycerides.

### Functional analysis of the *HMGA1* rs139876191

**Figure 1:**
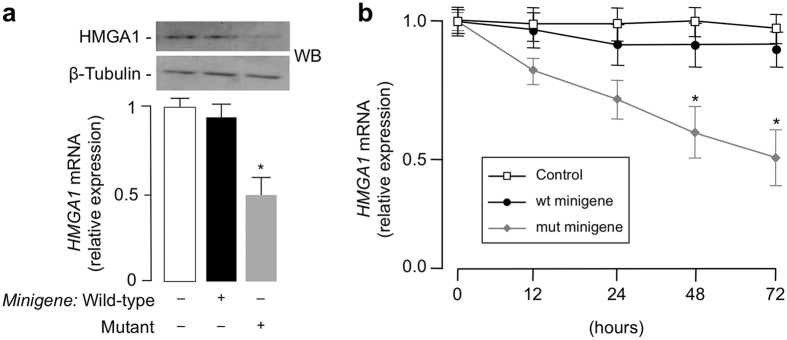
Effect of the *HMGA1* minigene on HMGA1 expression. (**a**) HEK-293 cells were transiently transfected with 1 μg wild-type or mutant minigene construct, and endogenous HMGA1 mRNA and protein levels were measured 72 h later by qRT-PCR and Western blot (WB), respectively. Cropped blots are shown in the figure. Full-length WBs are presented in Supplementary Fig. S1. *P < 0.05 vs control (white bar). β-tubulin, control of protein loading. (**b**) Time course of HMGA1 mRNA abundance in HEK-293 cells, untreated (control, open square) or treated with wild-type (wt, solid circle) or mutant (mut, solid diamond) *HMGA1* minigene. *P < 0.05 vs control. Data are mean ± standard error of the mean (s.e.m) of three independent experiments, each in triplicate.

The influence of the rs139876191 variant on the functionality of *HMGA1* gene was analyzed using the minigene strategy, an approach that has the potential to evaluate the significance of intronic polymorphisms^24^. Using this strategy, we analyzed the impact of the rs139876191 variant at the HMGA1 mRNA and protein levels, as this was of crucial importance for evaluating the relevance of this mutation on gene expression. For these experiments, either wild-type or mutant minigenes were transfected in HEK-293 cells and endogenous HMGA1 mRNA was measured by qRT-PCR. As shown in Fig. 1a, HMGA1 mRNA was significantly reduced in cells transfected with the mutant minigene construct compared to cells transfected with the wild-type minigene, thus indicating that the rs139876191 variant is functional and exhibits a dominant negative effect over endogenous HMGA1. As shown in time-course experiments, the negative effect of the mutant minigene on endogenous HMGA1 mRNA expression was time-dependent, with maximal inhibition at 72 h post transfection (Fig. 1b). Interestingly, the magnitude of endogenous HMGA1 protein and mRNA reductions in HEK-293 cells transfected with the mutant *HMGA1* minigene was consistent with the reduction of HMGA1 expression previously observed in blood monocytes of diabetic patients with the rs139876191 variant^25^.

### Influence of the HMGA1 rs139876191 on serum cytokines and other serum factors

The role of inflammatory proteins, cytokines and adhesion molecules in the onset and progression of DR is well known^26^. As they may serve as potentially useful biomarkers for early detection and prognosis of DR, numerous studies have been conducted over the past years analyzing serum levels of these molecules in affected diabetic patients^27^,^28^. However, conflicting data have been published on this subject, mostly because of the many confounding factors, such as comorbid disorders, variability in glucose levels and drug intake, which may affect analytical results, thereby reducing the clinical significance of changes in serum levels of these markers. Because of these considerations and in order to explore the influence on serum cytokines, growth factors and adhesion molecules exerted by the *HMGA1* rs139876191, an apparently healthy (untreated/nonsmoker) group of Calabrian individuals was also included in this study and used to measure the serum levels of these circulating factors. Within this group of healthy subjects, we selected 37 carriers of the rs139876191 variant and 97 non-carriers, carefully matched for age, sex, BMI and FPG^29^. As shown in Table 4, fasting serum VEGFA levels were significantly lower in healthy subjects carrying the *HMGA1* rs139876191 compared to wild-type subjects (*P* = 0.019). No other associations were statistically significant, although we cannot exclude a type II error due to the small number of analyzed subjects (Table 4). Based on this association and on the established role of VEGFA in retinal neovascularization, it can be hypothesized that the reduced risk of developing PDR in type 2 diabetic patients carrying the rs139876191 polymorphism can be ascribed, at least in part, to the lower concentration of VEGFA serum levels seen in such patients, and this is consistent with the above experimental results *in vitro* with minigenes, showing a dominant negative effect of the rs139876191 variant over endogenous HMGA1 expression.

### HMGA1 regulates the expression of VEGFA

**Figure 2:**
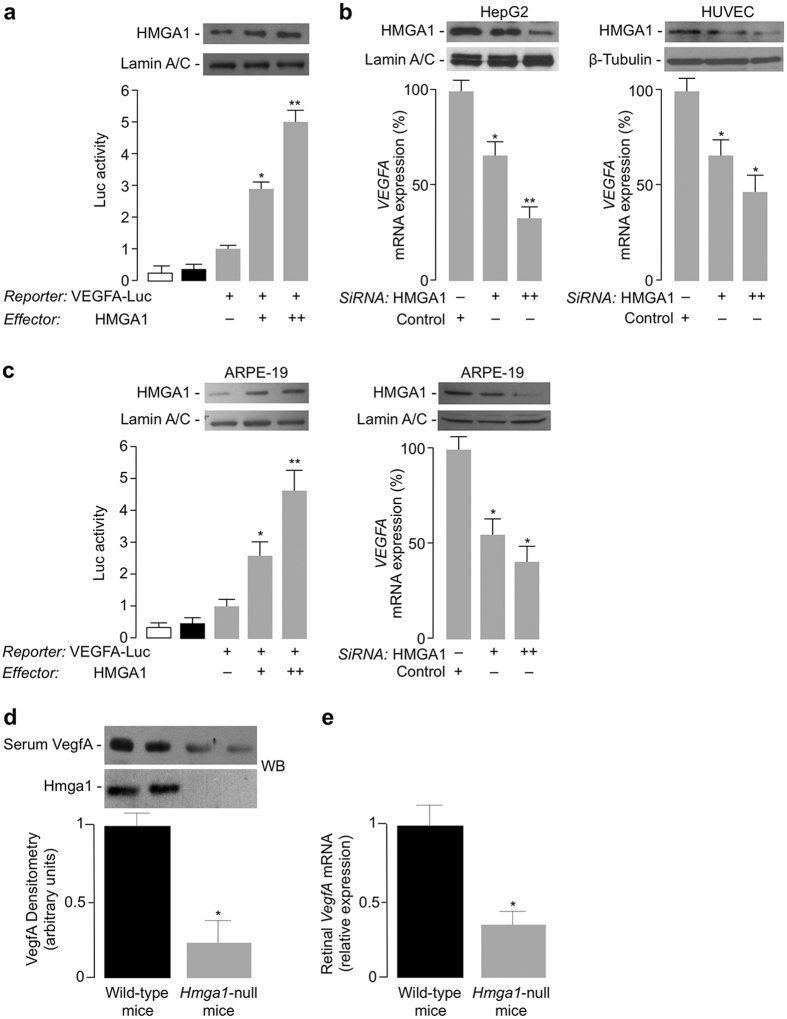
*VEGFA* gene expression is induced by HMGA1. (**a**) Human VEGFA-Luc reporter vector (2 μg) was transfected into HepG2 cells, in the presence of increasing amounts (0, 0.5, 1 μg) of HMGA1 effector plasmid, and Luc-activity was measured 48 h later. Data represent means ± s.e.m for three separate experiments; values are expressed as the factors by which Luc-activity increased above the level of the activity obtained in transfections with VEGFA-Luc reporter vector plus the empty effector vector (control), which is assigned an arbitrary value of 1. White bar, mock (no DNA); black bar, pGL3-basic (vector without an insert). *P < 0.05 and **P < 0.01 vs control. (**b**) qRT-PCR of endogenous VEGFA mRNA from HepG2 (left), and HUVEC (right) cells, pretreated with increasing amounts (100 and 200 pmol) of anti-HMGA1 siRNA or nontargeting control siRNA. (**c**) VEGFA-Luc-activity and qRT-PCR of endogenous VEGFA mRNA were measured in ARPE-19 cells, under the same conditions as in (a) and (**b**). WBs of HMGA1 in each condition are shown in the autoradiograms. Lamin A/C and β-Tubulin, controls of protein loading. Cropped blots are shown in the figures. Full-length WBs are presented in Supplementary Fig. S1. *P < 0.05 and **P < 0.001 vs siRNA-untreated (control) cells. (**d**) Representative VegfA WB of blood serum from wild-type and *Hmga1*-deficient mice. Densitometric analyses of six to eight independent blots are shown. Black bars, wild-type mice, n = 8; gray bars, *Hmga1*-knockout mice, n = 6. *P < 0.05 vs wild-type controls. Hmga1 protein expression is shown in fat tissue. All the samples were run under the same experimental conditions. Cropped blots are shown in the figures. Full-length WBs are presented in Supplementary Fig. S1. (**e**) VegfA mRNA levels in retinal tissue of wild-type (black bars) and *Hmga1*-deficient (gray bars) mice (n = 6 per genotype), as measured by qRT-PCR. Data are means ± s.e.m of three independent measurements from each animal. *P < 0.05 vs wild-type controls.

The relationship between HMGA1 and VEGFA was further investigated in cell transfection studies using reporter gene assays and confirmed *in vivo*, in *Hmga1*-knockout mice. As shown in Fig. 2a, transfection of HMGA1 expression vector significantly increased VEGFA luciferase activity in VEGFA-Luc-transfected HepG2 cells, and this effect occurred in a dose-dependent manner. Consistent with this, VEGFA mRNA levels were reduced in both HepG2 and HUVEC cells pretreated with siRNA targeting HMGA1 (Fig. 2b). These results were further supported by functional genomics data from ARPE-19 cells, a human retinal pigment epithelium cell line ideally suited for VEGF expression analysis^30^. As shown in Fig. 2c, VEGFA-Luc activity significantly increased in ARPE-19 cells overexpressing HMGA1, whereas VEGFA mRNA levels were decreased by siRNA targeting HMGA1, thus indicating that HMGA1 is required for proper transcription of the *VEGFA* gene. This conclusion was supported by the results obtained from *Hmga1*-knockout mice, in which, in line with findings in humans, serum levels of VEGFA were significantly lower when compared with normal wild-type mice (Fig. 2d). To exclude any discrepancy between serum and retinal VEGFA, and to corroborate data in humans, *VEGFA* gene expression was also investigated in retinal tissue from *Hmga1*-knockout mice. Consistent with the above results, VEGFA mRNA was significantly reduced in retina from mutant mice compared with wild-type controls (Fig. 2e).

### HMGA1 and hypoxia

**Figure 3:**
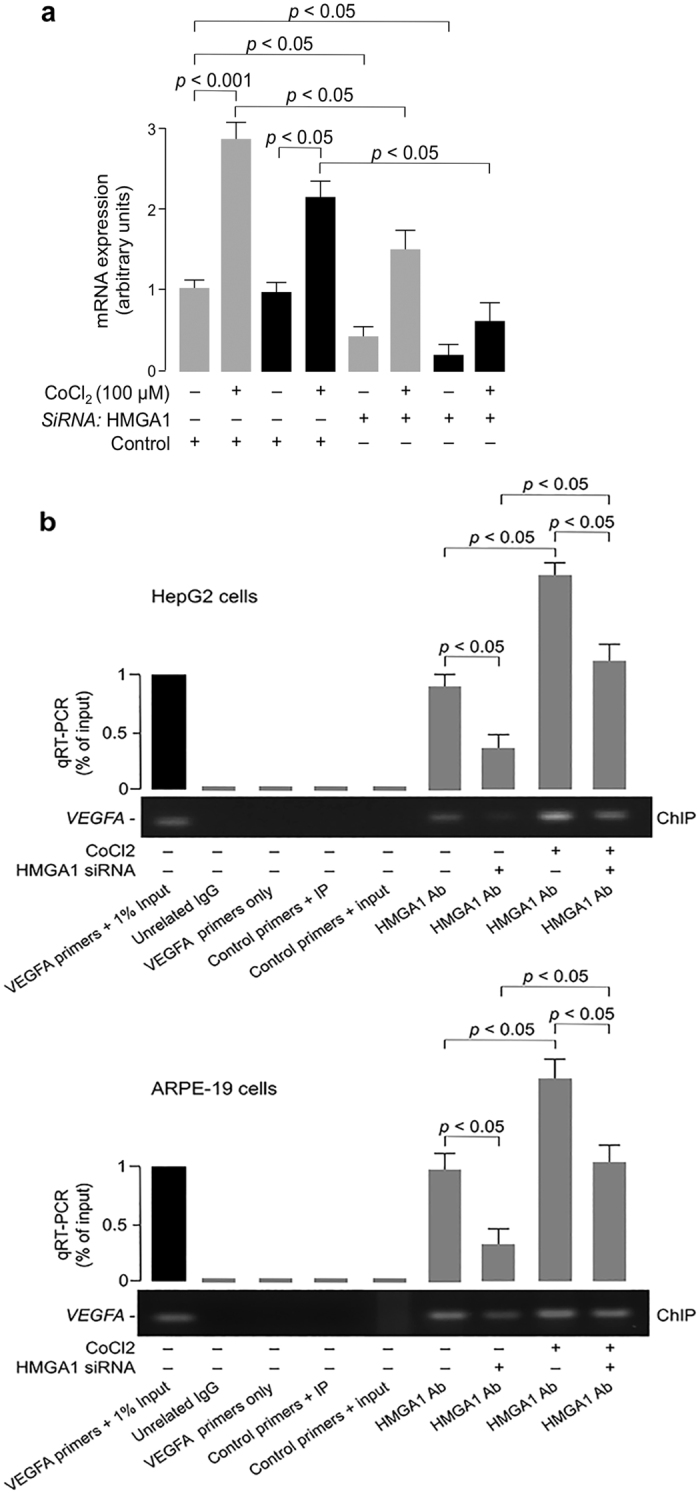
HMGA1 and VEGFA expression in hypoxia. (**a**) Effect of hypoxia on VEGFA (gray bars) and HMGA1 (black bars) mRNA, in HepG2 cells preatreated or not with anti-HMGA1 siRNA, as measured by qRT-PCR. Data are means ± s.e.m of three independent experiments, each performed in triplicate. (**b**) ChIP of the *VEGFA* promoter gene in HepG2 and ARPE-19 cells, either untreated or pretreated with siRNA against HMGA1, both in normoxic and hypoxic conditions, using an anti-HMGA1 specific antibody (Ab). Representative assays are shown, together with qRT-PCR of ChIP-ed samples. Cropped gels are shown in the figures. Full-length ChIPs are presented in Supplementary Fig. S1.

The promoting effect of hypoxia on VEGFA expression is well characterized^31^,^32^ and it may constitute a major trigger mechanism for VEGFA-induced neoangiogenesis in PDR^4^. Therefore, we finally evaluated the role of HMGA1 on VEGFA expression in hypoxia. For this purpose, normoxic HepG2 cells were exposed for 24 h to CoCl2, a known chemical hypoxic mimetic^33^, and VEGFA and HMGA1 mRNA levels were measured. As shown in Fig. 3a, VEGFA mRNA increased two-three fold in cells maintained in hypoxic conditions, and this increase paralleled the increase of HMGA1 mRNA abundance. However, when HMGA1-knockdown HepG2 cells were exposed to CoCl2, hypoxia-induced VEGFA and HMGA1 expressions were significantly blunted (Fig. 3a). These data were corroborated by ChIP-qPCR assays, showing that binding of HMGA1 to the endogenous VEGFA locus was considerably decreased in whole, intact HepG2 and ARPE-19 cells exposed to HMGA1 siRNA, either in normoxia or hypoxia (Fig. 3b), thus confirming the essential role of HMGA1 in VEGFA expression, and supporting the notion that a deficit of HMGA1 as that observed in type 2 diabetic patients with the *HMGA1* rs139876191 may protect against hypoxia-induced damages, including PDR, and perhaps other VEGFA-related diabetic complications.

## Discussion

Herein, we evaluated the association of the *HMGA1* rs139876191 with DR in an Italian cohort of type 2 diabetic patients. Our data indicate that the rs139876191 variant was present in 4.8% of patients with PDR compared with 8.3% of diabetic patients without PDR (controls plus NPDR), thus suggesting this variant may confer protection against PDR. Similarly to other studies in this series^34^, the protective effect of the *HMGA1* rs139876191 was confined to PDR only, with no effects on the susceptibility of NPDR. These findings were supported by subsequent studies *in vitro*, demonstrating the functional significance of the variant allele, which showed a dominant negative effect towards the wild-type allele. As for other similar reports referring to polymorphisms at intron/exon boundaries^24^, we can hypothesize that the rs139876191 variant could probably affect exonic or intronic splicing regulatory elements, resulting in skipping of exons or other alterations adversely affecting the encoded protein product. Based on the observation that serum VEGFA levels were lower in individuals carrying this variant than in those with the reference allele, it was tempting to hypothesize that the rs139876191 variant, by adversely affecting HMGA1 protein production, could have a role in this reduction. With this in mind, we have performed functional studies with the aim to provide a mechanistic explanation for the protective effect of the rs139876191 variant. Here we report for the first time that HMGA1 is a transcriptional regulator of VEGFA and that HMGA1 is required for the response of VEGFA to hypoxia. Therefore, although other factors in addition to or in concert with VEGFA may contribute to PDR, we can postulate that a deficit of HMGA1 in retinal tissues may protect against hypoxia-induced VEGFA-mediated neovascularization, at least in part through suppression of VEGFA expression. Some of the findings in humans were recapitulated in studies of *Hmga1*-knockout mice, in which VEGFA was considerably decreased in blood serum and retinal tissue, further supporting the concept that the rs139876191 variant decreases VEGFA expression.

To shed light on the genetic susceptibility to DR, extensive candidate gene studies and linkage analyses have been performed so far, and numerous genetic variants associated with DR have been identified in several genes, including the *VEGFA* gene^8^. Although much of these data are controversial, there is evidence that VEGFA plays a key role in the development of retinal neovascularization, and DR in particular. Our data herein well support this view and suggest that abnormalities in *VEGFA* gene regulation, which may be associated with decreased levels of VEGFA, might occur in patients with PDR, in the absence of mutations within the *VEGFA* locus.

One strength of the present work is the same ethnic origin of the subjects studied, which minimizes the risk of population stratification. Also, given that diabetic individuals may develop PDR later in life, it is easy to understand how important it can be to define the selection criteria for patients without PDR. In our study, in order to reduce this selection bias, diabetic subjects without PDR were recruited among patients with at least 10 years of duration of diabetes and this, in our opinion, constitutes a further strength of this work. Conversely, as the study design was confined to a single population, the lack of replication studies can be seen as a limitation, and the results unable to be generalized across populations. On the other hand, to our knowledge, there is no available genome-wide dataset on DR patients which includes the *HMGA1* rs139876191. Among the reasons for this omission, is probably the fact that typically GWAS are designed to exclude variants with a MAF < 5%^35^. Nevertheless, we want to point out that the present study is clearly not solely an association study between *HMGA1* rs139876191 and DR, but of particular interest is the simultaneous demonstration as to how this association translates pathophysiologically, providing a mechanistic explanation for the protective effect of this variant on DR.

Therefore, our findings may provide new insights into the molecular mechanisms underlying the development of PDR. Targeting HMGA1 function in *VEGFA* gene expression might be a novel approach for fighting VEGFA-dependent neovascularization and vascular permeability of retinal vessels. Also, because the *HMGA1* rs139876191 defines a specific defect that causes decreased VEGFA, diabetic patients with this variant may have a less severe clinical course of DR than other diabetic patients, and this could not only explain why patients may respond differently to a specific therapy, such as anti-VEGF medicines, but also provide an opportunity for designing tailored treatments based on patient genotype or marker expression. Confirmation of these results in other populations would be important to further validate our findings and their implications in a clinical setting.

## Methods

### Ethics statements

The study was approved by the local ethics committee, Regione Calabria Comitato Etico Sezione Area Centro (protocol registry n. 116 of May 14, 2015). All human participants gave written informed consent, and the methods were performed in accordance with approved guidelines. All animal work was conducted at the animal facility of the “Istituto Nazionale dei Tumori, Fondazione G. Pascale, Napoli”, using approved animal protocols, in accordance with relevant institutional guidelines for animal research (directive n. 86/609/ECC, European Community Council).

### Study cohort

We studied 1959 consecutive patients with type 2 diabetes, with and without DR, attending the Operative Units of Diabetes (Hospital Pugliese-Ciaccio, Catanzaro) and Endocrinology (University “Magna Græcia”, Catanzaro), between March 2008 and July 2014. All the study subjects were recruited in Calabria (Southern Italy), a region which consists of a population of comparatively limited genetic diversity^36^. First- and second-degree relatives were avoided on the basis of anamnestic data. Diabetes was diagnosed according to American Diabetes Association (ADA) criteria based on fasting glucose levels (≥126 mg/dL), or glucose levels following a 2 h OGTT (≥200 mg/dL). Patients with type 1 diabetes were excluded on clinical grounds and, in non-unequivocal cases, on the basis of plasma C-peptide levels and negative pancreatic islet-related autoantibodies. In all participants, the diagnosis of DR was based on ophthalmological examination of the ocular fundus after dilation of the pupils by experienced ophthalmologists. DR was graded according to the International Clinical Diabetic Retinopathy Disease Severity Scale^37^. Among the patients studied, 436 were classified as having PDR (retinal neovascularization and/or vitreous/preretinal hemorrhage), 587 had NPDR (microaneurysms, retinal hemorrhages, venous beading, retinal edema, hard exudates) and 936 patients were classified as controls with no retinal findings. Patients with pan-retinal photocoagulation were included in the PDR group and the severity of DR was graded based on the worst eye. Ophthalmological examination including fundus examination was carried out annually for all patients. To exclude misclassification due to the short duration of the disease, only patients with at least 10 years duration of diabetes were included in the control group.

### Anthropometric and biochemical measurements

A series of anthropometric and clinical parameters were evaluated for all patients. Fasting blood samples were collected and biochemical analyses of plasma glucose, triglycerides, total and HDL-cholesterol and serum insulin were performed in all participants with no caloric intake for at least 8 h. LDL-cholesterol was calculated using Friedewald’s formula. Serum concentration of 12 different cytokines and growth factors (IL-1α/IL-1β, IL-2, IL-4, IL-6, IL-8, IL-10, IFNγ, TNFα, MCP-1, VEGFA, EGF), as well as adhesion molecules (E-selectin, P-selectin, L-selectin, VCAM1, ICAM1) was simultaneously determined in a substudy of 134 matched healthy subjects^29^, either carriers or non-carriers of the rs139876191 allele, in the absence of drug treatments, using the biochip analyser Evidence Investigator (Randox Labs).

### Genotyping of the *HMGA1* rs139876191 and its functional analysis

Genomic DNA was extracted from peripheral blood and genotyped for rs139876191 by the fluorescence-based TaqMan allelic discrimination technique (Applied Biosystems)^25^. Reliability of the TaqMan detection method was confirmed by sequencing analysis of 160 DNA samples from diabetic patients with PDR, 68 DNA samples from patients with NPDR, and 72 DNA samples from control individuals (concordance rate >99%), which were directly sequenced for the exon 6 and adjacent introns of the *HMGA1* gene (NC_000006.11, http://www.ncbi,nlm.nih.gov). To clarify the functional relevance of the rs139876191 polymorphism (also designated IVS5–13insC), normal and mutant minigenes, spanning exons 5 to 6 of the human *HMGA1* gene, which included the C insertion site, were constructed (GenScript) and transiently transfected in HEK-293 cells naturally expressing HMGA1. Cell transfection efficiency was assessed by evaluating the expression levels of the minigenes with real-time PCR using internal primers (for 5′–ATCACTCTTCCACCTGCTCCTTA–3′; rev 5′–TTAGGTGTTGGCACTTCGCTG–3′), whereas endogenous *HMGA1* mRNA was measured by using primers external to the minigenes^38^. *RPS9* cDNA was used as an internal standard. PCR reactions were run in triplicate.

### Cell cultures, transfection, and ChIP

HepG2 human hepatoma cells, HEK-293 cells, and ARPE-19 cells were cultured as described previously^30^,^39^,^40^. HUVEC cells (Clonetics) were grown in endothelial cell growth medium 199 with 15% FBS, 2 mM glutamine, 100 μg/ml penicillin/streptomycin, heparin (100 μg/ml) and endothelial growth factors (30 μg/ml), and maintained in a 37 °C incubator in 5% CO2. Activity of the human VEGFA promoter construct pGL3-2.6 (a gift from Dr. Sheehy, University College Dublin, Ireland) was assessed in HepG2 and ARPE-19 cells, in the absence or presence of HMGA1 expression plasmid^40^. Luciferase activity was assayed 48 h after transfection, using the dual-luciferase reporter assay system (Promega)^41^. For gene silencing experiments, human anti-HMGA1 siRNA (Santa Cruz Biotech) was used. Transfections were done using Lipofectamine 2000 (Invitrogen). ChIP was performed in cells, pretreated with HMGA1 siRNA or control siRNA, as described^42^. Sequence-specific primers for *VEGFA* gene promoter used for PCR and qRT-PCR were: for 5′–TGGACCGGTCAGCGGACTCAC–3′; rev 5′–GCTCTCTCTGACCCCGTCTCT–3′.

### Studies in animals

Male *Hmga1*-deficient and wild-type mice aged 6–9 months were studied. Generation of these animals has been discussed in detail^13^. Blood was collected from the retro-orbital sinus of each mouse, and serum VEGFA levels measured by Western blot (WB), using an anti-VEGFA specific antibody (Santa Cruz Biotech). For qRT-PCR, total RNA was extracted from retinas obtained from the different groups of animals and cDNAs were synthesized using the RETROscript first strand synthesis kit (Ambion). Primers for mouse VEGFA (NM_019739): 5′-CAAAGTACACATACGGCCAATCC-3′; 5′-CGTAACTTGATTTGCTGTCCTGAA-3′.

### Statistical analysis

Initially, each quantitative trait was tested for normality of distribution (Shapiro-Wilk’s test) and log-transformed when required. Continuous variables are expressed as median and interquartile range (IQR), and categorical variables as number and percentage. The non-parametric Mann-Whitney test was used for comparisons of continuous variables between two groups, whereas the 2-tailed Fisher exact test was used for comparisons of proportions. In all analyses, a significance level of 0.05 was chosen. Logistic regression analysis was used to assess the independent role of the rs139876191 variant as possible predictor of DR, providing odds ratios (OR) with 95% confidence bounds. Instead, linear regression analysis was employed to find out the quantitative variables that were independently associated with this variant. In both cases, appropriate covariates were added. Because of the low frequency of the rs139876191 variant, only the dominant genetic model was considered. Data were analyzed with SPSS Version 20.

## Additional Information

**How to cite this article**: Chiefari, E. *et al*. A polymorphism of *HMGA1* protects against proliferative diabetic retinopathy by impairing HMGA1-induced VEGFA expression. *Sci. Rep.* **6**, 39429; doi: 10.1038/srep39429 (2016).

**Publisher's note:** Springer Nature remains neutral with regard to jurisdictional claims in published maps and institutional affiliations.

## Supplementary Material

Supplementary Information

## Figures and Tables

**Table 1:** Clinical and biochemical baseline characteristics of enrolled patients.

	Control n = 936	NPDR n = 587	PDR n = 436
Ethnicity	Caucasian	Caucasian	Caucasian
Female (%)	505 (54.0)	282 (48.0) [0.027]	212 (48.6) [0.072]
Age (yr)	66 (61–73)	66 (61–72) [0.926]	67 (60–73) [0.374]
Age at onset of diabetes (yr)	52 (46–58)	51 (47–58) [0.748]	51 (46–58) [0.499]
Duration of diabetes (yr)	13 (11–18)	14 (11.5–17) [0.476]	15 (11–18) [0.607]
Family history of diabetes	582 (62.2)	374 (63.7) [0.550]	280 (64.2) [0.472]
BMI (Kg/m^2^)	27.9 (26.1–30.2)	27.8 (26.0–30.5) [0.727]	27.4 (26.0–30.0) [0.210]
Systolic BP (mmHg)	130 (125–140)	135 (125–140) [<0.001]	135 (125–145) [< 0.001]
Diastolic BP (mmHg)	80 (70–80)	80 (75–80) [0.053]	80 (75–80) [< 0.001]
Hypertension (140/90)	499 (53.3)	328 (55.9) [0.342]	280 (64.2) [< 0.001]
Antihypertensive therapy (n)	325 (34.7)	218 (37.1) [0.350]	178 (40.8) [0.030]
FPG (mg/dL)	158 (150–176)	159 (150–180) [0.984]	165 (150–181) [0.004]
HbA1c (%)	7.6 (7.2–8.2)	7.6 (7.3–8.4) [0.755]	7.8 (7.3–8.5) [0.002]
HbA1c (mmol/mol)	60 (55–66)	60 (56–68)	62 (56–69)
Diet treatment alone (n)	3 (0.3)	1 (0.2) [0.999]	2 (0.5) [0.656]
Non-insulin hypoglycemic agents only (n)	527 (56.3)	288 (49.1) [0.006]	131 (30.0) [< 0.001]
Insulin therapy (n)	406 (43.3)	298 (50.8) (0.005)	303 (69.5) [< 0.001]
Total cholesterol (mg/dL)	169.0 (151.0–190.0)	169.0 (149.0–190.0) [0.937]	169.0 (149.3–192.0) [0.942]
HDL-C (mg/dL)	47.0 (43.0–52.0)	46.0 (40.0–55.0) [0.116]	46.0 (40.0–54.0) [0.052]
LDL-C (mg/dL)	94.2 (76.4–114.6)	98.0 (76.1–113.6) [0.982]	94.8 (78.2–114.9) [0.969]
Triglycerides (mg/dL)	125 (99–158)	125 (99–158) [0.613]	123 (95–160) [0.300]
Hypolipidemic therapy (%)	405 (43.3)	329 (56.0) [<0.001]	272 (62.4) [< 0.001]
Creatinine (mg/dL)	1.1 (1.0–1.2)	1.0 (0.9–1.2) [0.127]	1.1 (0.9–1.2) (0.094)
Macroangiopathy (n)^a^	216 (23.1)	144 (24.5) [0.536]	114 (26.1) [0.223]
Nephropathy (n)^b^	105 (11.2)	79 (13.4) [0.197]	70 (16.1) [0.015]
Foot disease (n)^c^	195 (20.8)	134 (22.8) [0.338]	111 (25.5) [0.052]

Data are medians (IQR) or n (%). Non-parametric Mann-Whitney test was used for distribution comparisons of quantitative variables. The two-tailed Fisher Exact Test was used for proportion comparisons between groups. *P* values versus control patients without DR are shown in square brackets. Significance level < 0.05. BMI, body mass index; BP, blood pressure; FPG, fasting plasma glucose; HDL-C, high-density lipoprotein-cholesterol; LDL-C, low-density lipoprotein-cholesterol.

^a^Includes myocardial infarction, coronary heart disease, and stroke.

^b^Refers to people with urine albumin:creatinine ratio >30 μg/mg, or with albumin excretion rate >30 mg/day (ADA criteria).

^c^Includes foot ulceration, lower-extremity amputation, and sensory impairment.

**Table 2:** Association of the *HMGA1* rs139876191 variant with DR.

	–/– (%)	–/C (%)	C/C (%)	MAF (%)	^a^Adjusted OR (95% CI)	^a^Adjusted *P*-value	^b^Adjusted OR (95% CI)	^b^Adjusted *P*-value
PDR	415 (95.2)	20 (4.6)	1 (0.2)	2.52	0.573 (0.348–0.943)	0.029	0.5183 (0.309–0.868)	0.013
Controls	861 (92.0)	75 (8.0)	0 (0)	4.01				
NPDR	537 (91.5))	50 (8.5)	0 (0)	4.26	1.0633 (0.731–1.546)	0.748	1.0463 (0.716–1.528)	0.816
Controls	861 (92.0)	75 (8.0)	0 (0)	4.01				

Logistic regression analysis was performed to assess the independent role of the rs139876191 variant on PDR and NPDR. Adjusted OR and P values are shown. ^a^Age and sex or ^b^age, sex, duration of diabetes, hypertension (systolic BP ≥ 140 mmHg and/or diastolic BP ≥ 90 mmHg), antihypertensive therapy, HbA1c, HDL cholesterol, and hypolipidemic therapy were added as covariates.

**Table 3:** Clinical and biochemical traits in the study population, according to the presence of the rs139876191 variant.

Population	Carrier	Wild-Type	*P-*value
n	146	1823	
Age (yr)	65.5 (60–71.25)	66 (61–73)	0.417
Female (n)	69 (47.3)	941 (51.6)	0.263
Age at onset of diabetes	52 (47.8–58)	51 (47–58)	0.715
BMI (Kg/m^2^)	28.0 (26.0–31.1)	27.8 (26.0–30.2)	0.309
Systolic BP (mmHg)	135 (125–140)	130 (125–140)	0.878
Diastolic BP (mmHg)	80 (75–80)	80 (70–80)	0.688
Hypertension (n)	84 (57.5)	1023 (56.4)	0.862
FPG (mg/dL)	161 (150–184)	160 (150–178)	0.118
HbA1c (%)	7.9 (7.1–8.7)	7.7 (7.2–8.3)	0.096
HbA1c (mmol/mol)	63 (54–72)	61 (55–67)	
Total cholesterol (mg/dL)	168.5 (152.0–191.0)	169.0 (150.0–190.0)	0.785
HDL-C (mg/dL)	47.0 (40.0–51.0)	47.0 (42.0–54.0)	0.150
LDL-C (mg/dL)	96.6 (81.1–116.3)	94.6 (76.0–114.0)	0.284
Triglycerides (mg/dL)	122 (100–160)	125 (98–158)	0.850
Creatinine (mg/dL)	1.1 (0.9–1.2)	1.1 (1.0–1.2)	0.991
Macroangiopathy (n)^a^	41 (28.1)	435 (23.8)	0.423
Microangiopathy (n)	37 (25.3)	515 (28.3)	0.847
Nephropathy (n)^b^	13 (8.9)	241 (13.2)	0.249
Foot disease (n)^c^	37 (25.3)	408 (22.3)	0.680

Data are medians (IQR) or n (%). Quantitative traits were log-transformed to better approximate a normal distribution and after compared using linear regression analysis adjusted for age, gender (and BMI when appropriate). The two-tailed Fisher Exact Test was used for proportion comparisons between groups. Significance level <0.05. BMI, body mass index; BP, blood pressure; FPG, fasting plasma glucose; HDL-C, high-density lipoprotein-cholesterol; LDL-C, low-density lipoprotein-cholesterol.

^a^Includes myocardial infarction, coronary heart disease, and stroke.

^b^Refers to people with urine albumin:creatinine ratio >30 μg/mg, or with albumin excretion rate >30 mg/day (ADA criteria).

^c^Includes foot ulceration, lower-extremity amputation, and sensory impairment.

**Table 4:** Serum cytokines profile and other serum factors in a healthy population, according to the presence of the rs139876191 variant.

	Carrier n = 37	Non-carrier n = 97	*P-*value
Age (yr)	54 (47.5–61.5)	56 (48.5–60.0)	0.998
Female	18 (48.6)	47 (48.5)	0.999
BMI (Kg/m^2^)	24 (22.9–25.3)	24.3 (23.1–25.1)	0.901
Systolic BP (mmHg)	122 (117–130)	122 (120–130)	0.781
Diastolic BP (mmHg)	75 (70–80)	70 (70–80)	0.309
FPG (mg/dL)	88 (81.5–90)	82 (76.5–88)	0.774
IL1α (pg/mL)	0.0 (0.0–0.3)	0.0 (0.0–0.3)	0.651
IL1β (pg/mL)	0.0 (0.0–1.0)	0.0 (0.0–1.1)	0.413
IL2 (pg/mL)	3.0 (1.9–3.8)	3.3 (2.2–5.7)	0.079
IL4 (pg/mL)	1.5 (1.3–1.8)	1.3 (1.1–1.6)	0.112
IL6 (pg/mL)	1.4 (1.2–2.0)	1.2 (0.9–2.1)	0.136
IL8 (pg/mL)	2.4 (1.7–4.3)	2.6 (1.7–4.8)	0.356
IL10 (pg/mL)	0.5 (0.0–1.1)	0.7 (0.0–1.3)	0.346
VEGF (pg/mL)	31.9 (28.1–40.7)	35.5 (31.6–45.3)	0.019
IFNγ (pg/mL)	0.9 (0.4–2.9)	1.0 (0.4–2.9)	0.753
TNFα (pg/mL)	1.6 (1.2–2.2)	1.8 (1.4–2.4)	0.110
MCP1 (pg/mL)	178.9 (142.9–232.0)	212.5 (158.5–243.9)	0.197
EGF (pg/mL)	15.8 (9.5–31.4)	21.1 (12.9–30.9)	0.442
VCAM1 (pg/mL)	548.9 (439.3–638.4)	567.8 (462.3–640.5)	0.519
ICAM1 (pg/mL)	240.5 (219.1–273.9)	248.2 (221.7–279.1)	0.624
E selectin (pg/mL)	13.4 (10.1–17.1)	15.0 (11.2–18.8)	0.123
P selectin (pg/mL)	99.0 (90.1–122.6)	107.2 (92.8–129.0)	0.126
L selectin (pg/mL)	938.4 (825.9–1202.0)	957.3 (844.3–1236.0)	0.430

Data are medians (IQR) or n (%). Non-parametric Mann-Whitney test was used for distribution comparisons of quantitative variables. The two-tailed Fisher Exact Test was used for proportion comparisons between groups. Significance level < 0.05. BMI, body mass index; FPG, fasting plasma glucose; BP, blood pressure; IL, interleukin; VEGF, vascular endothelial growth factor; IFN-γ, interferon-gamma; TNF-α, tumor necrosis factor-alpha; MCP-1, monocyte chemoattractant protein-1; EGF, epidermal growth factor; VCAM1, vascular cell adhesion molecule 1; ICAM1, intercellular adhesion molecule 1.
