# Supplementary material for: A polymorphism of HMGA1 protects against proliferative diabetic retinopathy by impairing HMGA1-induced VEGFA expression
Source: Sci Rep. 2016 Dec 19;6:39429. doi: 10.1038/srep39429 (PMC5171873; doi:10.1038/srep39429)
Supplement: Supplementary Information [file srep39429-s1.pdf]

A polymorphism of *HMGA1* protects against proliferative diabetic retinopathy by impairing HMGA1-induced VEGFA expression

Eusebio Chiefari[1], Valeria Ventura[1], Carmelo Capula[2], Giorgio Randazzo[3], Vincenzo Scorcia[3], Monica Fedele[4], Biagio Arcidiacono[1], Maria Teresa Nevolo[1], Francesco Luciano Bilotta[1], Michela Vitiello[4], Camillo Palmieri[3], Elio Gulletta[1], Alfredo Fusco[4,5], Daniela Foti[1], Raffaella Vero[2] &

Antonio Brunetti[1]*

Supplementary Information

Supplementary Figure

Figure 1A

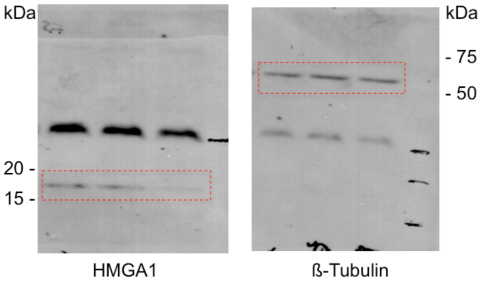

Figure 2A

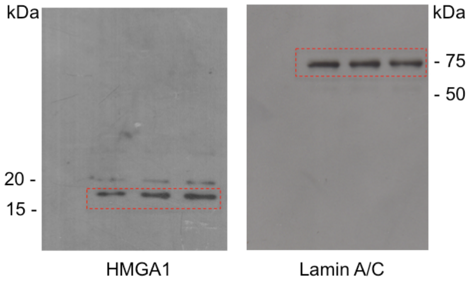

Figure 2B
HepG2

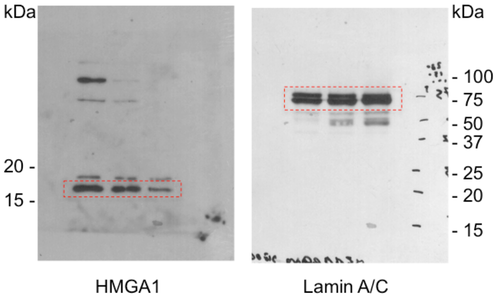

Figure 2B
HUVEC

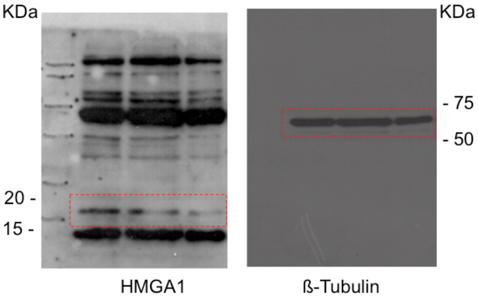

Figure 2C (left panels)

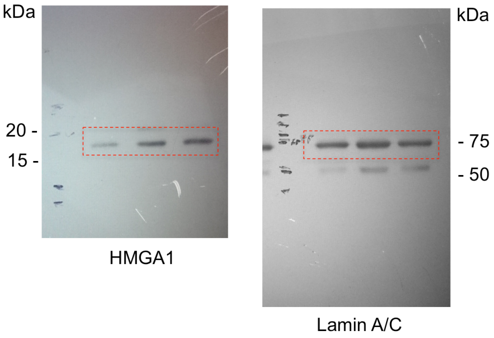

Figure 2C (right panels)

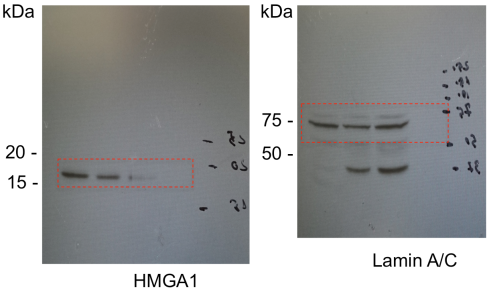

Figure 2D

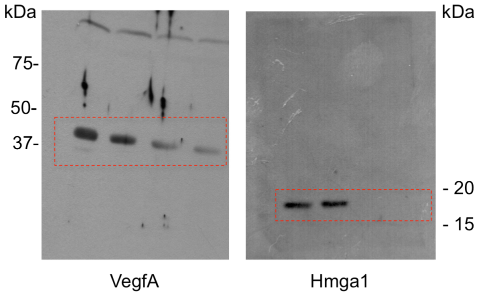

Figure 3B (upper panel)

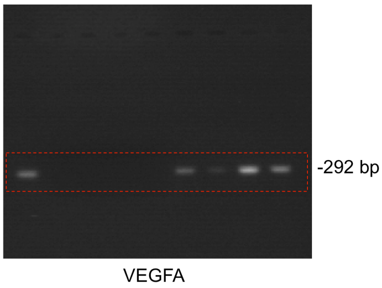

Figure 3B (lower panel)

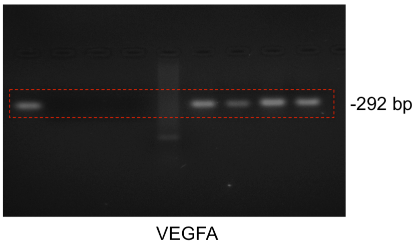

Supplementary Fig. S1: Full-length Western blots and gels. Western blots: Figure 1A, HMGA1, β-Tubulin; Figure 2A, HMGA1, Lamin A/C; Figure 2B (HepG2), HMGA1, Lamin A/C; Figure 2B (HUVEC), HMGA1, β-Tubulin; Figure 2C (left, ARPE-19), HMGA1, Lamin A/C; Figure 2C (right, ARPE-19), HMGA1, Lamin A/C; Figure 2D, VegfA, Hmga1. ChIP: Figure 3B, ChIP (HepG2) VEGFA; Figure 3B, ChIP (ARPE-19) VEGFA.
